# The critical role of artificial intelligence and bioinformatics in accelerating peptide-based vaccine discovery for tackling global infectious diseases

**DOI:** 10.1093/bib/bbag260

**Published:** 2026-07-17

**Authors:** Nomathamsanqa Tholo, Gavin Markey, Ruairidh Harrigan, Preeti Pandey, Bodhayan Prasad, Ram Shankar Barai, David Samuel Gibson, Priyank Shukla

**Affiliations:** Personalised Medicine Centre, School of Medicine, Ulster University, C-TRIC Building, Altnagelvin Area Hospital, Glenshane Road, Londonderry BT47 6SB, United Kingdom; Personalised Medicine Centre, School of Medicine, Ulster University, C-TRIC Building, Altnagelvin Area Hospital, Glenshane Road, Londonderry BT47 6SB, United Kingdom; Personalised Medicine Centre, School of Medicine, Ulster University, C-TRIC Building, Altnagelvin Area Hospital, Glenshane Road, Londonderry BT47 6SB, United Kingdom; Department of Genetics and Biochemistry, Clemson University, 190 Collings St., Clemson, SC 29634, United States; Wolfson Wohl Cancer Research Centre, School of Cancer Sciences, University of Glasgow (Garscube Campus), Glasgow G61 1QH, United Kingdom; Biological Sciences Division, ICMR - National Institute of Occupational Health, Meghani Nagar, Ahmedabad 380016, Gujarat, India; Personalised Medicine Centre, School of Medicine, Ulster University, C-TRIC Building, Altnagelvin Area Hospital, Glenshane Road, Londonderry BT47 6SB, United Kingdom; Personalised Medicine Centre, School of Medicine, Ulster University, C-TRIC Building, Altnagelvin Area Hospital, Glenshane Road, Londonderry BT47 6SB, United Kingdom

**Keywords:** artificial intelligence, machine learning, bioinformatics, immunoinformatics, peptide, vaccine

## Abstract

Peptide-based vaccines, enabled by bioinformatics and machine learning (ML), have emerged as one of the most promising approaches for rapid, safe, and cost-effective vaccine design against infectious diseases. Unlike conventional approaches that depend heavily on whole-pathogen cultures or recombinant protein expression, peptide vaccines can be designed *in silico* and synthesized quickly. Rational and targeted *in silico* approaches for the discovery of peptide-based vaccine candidates include B-cell and T-cell epitope prediction, immunogenicity, antigenicity, allergenicity, autoimmunity, population coverage, sequence conservation, molecular docking, molecular dynamics simulation, *in silico* cloning, and immunological simulation analyses. The combination of these comprehensive computational methods can effectively generate high-quality vaccine candidates for subsequent validation via *in vitro* and *in vivo* experiments. This review contextualizes the historical trajectory of peptide-based vaccinology, from early linear epitope discoveries in the 1960s to multi-epitope constructs and clinically tested candidates such as UB-612 and PepGNP-Covid19. It examines critical challenges in immunoinformatics, including performance gaps in epitope prediction tools, complexities in human leucocyte antigen (HLA) mapping, and the need for extensive manual intervention in pipelines. Artificial intelligence-driven approaches, spanning deep learning, and interpretable ML, are positioned to transform epitope prediction, reduce human error, and standardize reproducibility. These advances have the potential to support global outbreak response targets such as the Coalition for Epidemic Preparedness Innovations (CEPI) 100 Days Mission and the World Health Organization (WHO) Research and Development (R&D) Blueprint. However, their performance remains constrained by data quality, dataset imbalance, limited benchmark standardization, and persistent underrepresentation of many HLA alleles and population groups.

**Key Points**

- Peptide-based vaccines, accelerated by bioinformatics and machine learning, offer a potentially rapid, relatively safe, and cost-effective alternative to traditional vaccine design, enabling *in silico* development and swift synthetic manufacturing.
- Computational methods such as B-cell and T-cell epitope prediction, immunogenicity analysis, and molecular simulations allow for rational and targeted vaccine candidate discovery, enhancing quality and efficiency.
- The field has evolved from early linear epitope discoveries in the 1960s to sophisticated multi-epitope constructs and clinically tested candidates like UB-612 and PepGNP-Covid19.
- Major challenges in immunoinformatics include performance limitations in epitope prediction tools, complexities in HLA mapping, and the necessity for manual intervention in data pipelines.
- Artificial intelligence-driven models, including deep learning and interpretable machine learning, promise to overcome these challenges by improving prediction accuracy, reducing errors, and supporting global epidemic response efforts such as CEPI’s 100 Days Mission and the WHO R&D Blueprint.

## Introduction

**Figure 1:**
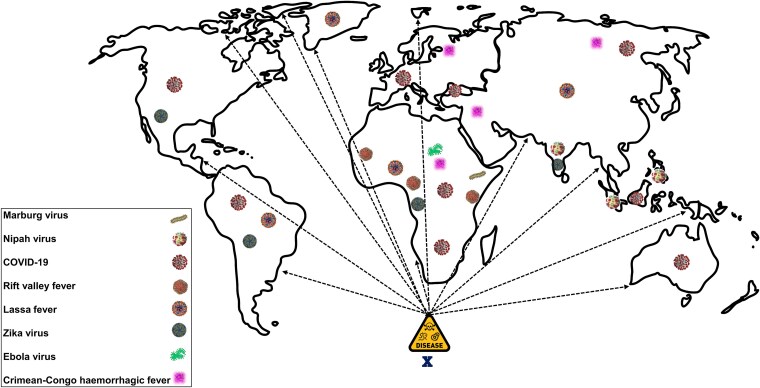
An overview of the current global infectious disease threats. The high public health risk diseases for research and development according to World Health Organization’s priority list [12]. The world map is from openclipart.org/173525 (CC-0 license).

**Figure 2:**
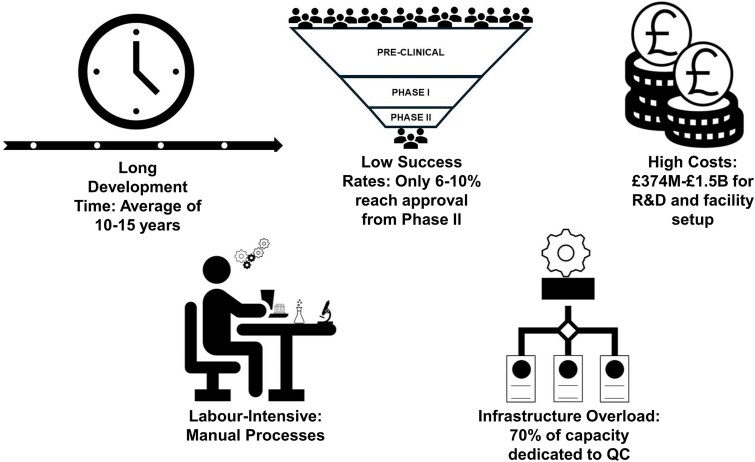
Traditional vaccine development problem overview. Conceptual summary of major bottlenecks associated with conventional vaccine development, including long development timelines, labour-intensive experimental workflows, high attrition rates, substantial infrastructure requirements, and high financial cost. The figure is designed to illustrate why early-stage antigen and epitope prioritization has increasingly shifted toward computational support, particularly for outbreak-prone pathogens where manual, sequential discovery processes are poorly matched to rapid-response needs [13, 14].

Vaccination is consistently recognized as one of the most effective public health measures, annually preventing millions of deaths from emerging and re-emerging infectious diseases (Fig. 1) such as smallpox, measles, and diphtheria [1, 2]. COVID-19 pandemic revealed critical challenges associated with conventional timelines for vaccine discovery and development [3]. Classical interventions involving heat-inactivated, chemically killed or live-attenuated pathogens [4–6] are expensive and inefficient, on average taking between 15 and 20 years to establish an effective vaccine candidate (Fig. 2), which is unfeasibly long when dealing with a growing number of novel endemic and pandemic level threats [7, 8]. The groundbreaking success of messenger ribonucleic acid (mRNA) vaccines illustrated that platform technologies with computational design tools can reduce the timeline to less than a year [9]. Peptide-based vaccines are a promising alternative, being synthetically manufacturable, thermally stable, cost effective, with a wide range of *in silico* tools available for identification and screening of promising candidates [10, 11].

Peptide-based vaccines incorporate short sequences of amino acids that contain B lymphocyte (B-cell) and T lymphocyte (T-cell) epitopes, which can be predicted from pathogenic proteomic data following sequencing, using a wide variety of immunoinformatic tools [15]. This approach allows immune responses to be targeted more precisely than in whole-pathogen or recombinant protein-based vaccines which include non-helpful antigenic constituents, reducing the risk of potentially unwanted side-effects [16]. Modern epitope prediction pipelines have begun to incorporate artificial intelligence (AI) and machine learning (ML)-based approaches in conjunction with traditional immunoinformatic pipelines, improving predictive accuracy and efficiency in peptide-based vaccine design [17, 18]. Complimentary tools in current pipelines ensure that predicted vaccine candidates can have important features including population coverage and safety profiles (allergenicity, autoimmunity, toxicity, etc.) assessed prior to *in vitro* and *in vivo* analysis [19, 20].

To position this review within the existing literature, a scoping search of PubMed and Google Scholar was undertaken using combinations of terms including ‘artificial intelligence,’ ‘machine learning,’ ‘immunoinformatics,’ ‘peptide vaccine,’ ‘epitope prediction,’ ‘vaccine design,’ and ‘review.’ Relevant reviews were selected based on their focus on peptide-vaccine immunoinformatics, computational vaccine design workflows, AI or machine learning applications, benchmarking, or translational discussion. As summarized in Table 1, prior reviews have typically focused on selected components of the field, such as epitope prediction, reverse vaccinology, or artificial intelligence applications, but have rarely integrated the historical evolution of peptide-vaccine immunoinformatics with data-resource limitations, reproducibility standards, human leucocyte antigen (HLA) equity, benchmarking challenges, and translational readiness in a single synthesis. This review addresses that gap by critically connecting these dimensions across the full design-to-translation pipeline.

**Table 1:** Comparative overview of review articles over the past decade relevant to immunoinformatics, epitope prediction, reverse vaccinology, and AI-enabled vaccine design, summarizing each review’s primary focus and main value.

**Authors**	**Year**	**Review method**	**Purpose of the study**
Backert and Kohlbacher [17]	2015	Narrative review	To review epitope prediction tools and demonstrate how immunoinformatics supports antigen and epitope discovery within genomic medicine.
Singh *et al.* [21]	2016	Narrative review	To synthesize available MHC-linked databases and prediction tools and evaluate their utility in vaccine-related antigen screening.
Dhanda *et al.* [22]	2017	Narrative review	To outline computational tools used to design peptide-based subunit vaccines and Immunotherapeutics.
De Brito *et al.* [23]	2018	Narrative review	To examine the rationale, targets, and challenges of peptide vaccine development for leishmaniasis.
Sirugo et al. [24]	2019	Narrative review	To highlight the underrepresentation of diverse populations in genetic studies and its implications for biomedical research.
Malonis *et al.* [25]	2019	Narrative review	To review progress, advantages, and ongoing challenges in peptide-based vaccine development.
Oli *et al.* [26]	2020	Narrative review	To provide a broad overview of how immunoinformatics contributes to vaccine development.
Kardani *et al.* [27]	2020	Narrative review	To demonstrate *in silico* vaccine design strategies applied across different pathogens and cancer.
Ortega-Tirado *et al.* [28]	2020	Narrative review	To review immunoinformatics approaches for T-cell peptide-based vaccine design against *Mycobacterium tuberculosis*.
Parvizpour et al. [29]	2020	Narrative review	To provide an overview of bioinformatics workflows used in epitope-based vaccine design.
Lim *et al.* [30]	2021	Narrative review	To discuss computational development of multi-epitope peptide vaccines against SARS-CoV-2.
Michalik *et al.* [31]	2021	Narrative review	To update the reverse-vaccinology field and establish connections between genomic data and computational epitope prediction for vaccine candidate selection.
Yurina *et al.* [32]	2022	Narrative review	To highlight the importance of *in silico* approach in vaccine development.
Cia *et al.* [33]	2023	Critical review	To critically evaluate methods for conformational B-cell epitope prediction.
Kumar *et al.* [34]	2023	Narrative review	To discuss conceptual and technical challenges in identifying B-cell epitopes.
Wei *et al.* [35]	2025	Narrative review	To review computational advances and tools evolution supporting multi-epitope vaccine development.
Kumar *et al.* [36]	2024	Narrative review	To review AI-enabled approaches for personalized cancer vaccine design.
Carroll *et al.* [37]	2024	Narrative review	To provide a method-oriented overview of structure-based and computational strategies for conformational B-cell epitope prediction.
Villanueva-Flores *et al.* [18]	2025	Narrative/comparative review	To systematically compare AI-driven epitope prediction approaches and provide practical guidance for vaccine development.
El Arab *et al.* [38]	2025	Systematic review	To comprehensively examine how artificial intelligence is being applied across multiple stages of vaccine research and development.

This review discusses how AI and bioinformatics are reshaping peptide-based vaccine discovery, examining the historical transition from traditional vaccine design towards computational approaches incorporating reverse vaccinology. We investigated current immunoinformatics strengths and the recent integration of AI in epitope prediction pipelines, alongside challenges that need to be addressed to improve pandemic preparedness and the goal of 100-day vaccine development [12, 13].

## Historical evolution of peptide-based vaccinology

### Early discoveries (1960s–1990s)

The original concept of peptide-based vaccines is based on the discovery that linear epitopes are functional minimal units of antigens. The first peptide vaccination study was reported by Anderer in 1963. A short hexapeptide fragment isolated from the tobacco mosaic virus (TMV) coupled to bovine serum albumin (BSA) induced TMV neutralizing antibodies in rabbits. This was the first clear evidence to support the use of a short peptide to mimic the native antigenic site and induce functional neutralizing antibodies [39]. In the preceding years, it was discovered that stimulation of immune responses by antigenic determinants (epitopes) is dependent on their recognition by complementary determining regions (CDRs), present on B and T-cell antigen receptors [40]. The discovery that epitopes are responsible for the specificity and strength of both humoral and cell-mediated immune responses formed the basis for strategies used in subsequent vaccine studies and development [41, 42].

**Figure 3:**
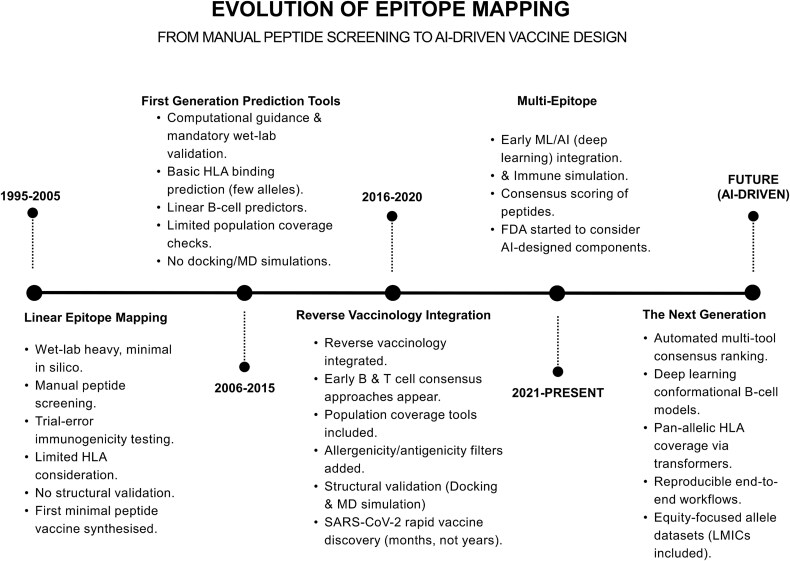
Historical evolution of peptide-based vaccine design from empiric immunology to integrated computational pipelines (1995 to present). The figure presents a schematic overview of the major conceptual shifts in peptide-based vaccine development across two decades. The early phase was dominated by wet-lab, trial-and-error immunology with limited computational input, minimal reproducibility, and little capacity for systematic prioritization of candidates [47]. The transitional phase introduced early sequence-based immunoinformatics and peptide–MHC binding prediction, allowing computational input for antigen selection but still with limited allele coverage, modest predictive performance, and continued dependence on laboratory screening [48]. The contemporary phase is characterized by reverse vaccinology, multistep *in silico* pipelines, consensus scoring across predictors, population coverage analysis, structural modelling, and the increasing use of machine learning and artificial intelligence to support candidate prioritization [1].

Clinical trials with mutant RAS and MUC-1 peptides in the 1990s showed that these vaccines were safe in humans, induced weak T-cell memory, both of which resulted in low clinical response [43, 44]. These failures highlighted key design needs; minimal epitopes failed to induce CD4^+^ T-helper interaction which is crucial for maintaining effective cytotoxic reactivity [45, 46]. This early landscape as summarized in Fig. 3 demonstrated a heavy dependence on trial-and-error immunology with minimal computational input or ability to predict functional leads [47].

### Transition to synthetic long peptides (2000s–2010s)

To overcome the low immunogenicity of minimal epitopes, synthetic long peptides (SLPs) and overlapped peptide pools were introduced. SLPs, which are generally 20–35 amino acids in length, need to be processed intracellularly by professional antigen-presenting (APC) cells to generate shorter epitopes for presentation to MHC molecules. This endogenous processing results in the induction of MHC class I epitopes (for stimulation of CD8^+^ cytotoxic T-lymphocytes) as well as MHC class II epitopes (to activate CD4^+^ helper T-cells), leading to B-cell activation, differentiation, and antibody production [48].

Concurrent improvements in adjuvant technology, such as Toll-like receptor (TLR) agonists and montanide emulsions, strongly improved T-cell priming and persistence [49, 50]. These developmental achievements have established that successful peptide vaccine formulations are in the realm of integrated design, which includes the selection of epitope, adjuvant, and delivery system. These developments entailed a move from weakly immunogenic minimal peptides to complex multi-epitope constructs capable of stimulating coordinated immune responses.

This era saw the first-generation of immunoinformatic tools like SYFPEITHI [51], BIMA [52] as well as early IEDB modules [53]. These tools allowed prediction of peptide–MHC binding affinities, but only for a limited set of alleles with moderate accuracy. From 2000 to 2010, the first computational approaches for vaccine design were incorporated into vaccine development workflows, as complements, rather than replacing to laboratory screening methods.

Between 1995 and 2015 (Fig. 3), vaccine design transitioned from empirical immunology [54] to semi-computational design, where computational models guided laboratory selections, despite limited consensus and poor reproducibility [29].

### Computational revolution (2010s–present)

The combination of reverse vaccinology and bioinformatics has accelerated computational epitope identification substantially shortening early-stage antigen and epitope prioritization timelines. However, translating computationally identified epitopes into clinically validated vaccines require extended experimental and regulatory pathways (months-years), which are not addressed by computational innovation alone [14, 55]. Earlier reviews have examined both the genuine advances and persistent bottlenecks in peptide vaccine design [29, 56].

In early 2020, the fast release of the SARS-CoV-2 protein sequence permitted direct use of reverse vaccinology technologies for faster vaccine development. Recent clinical candidates are proof that computationally identified epitopes can induce a robust immune response in people. The peptide vaccine for melanoma targeting the gp100 protein, demonstrated the clinical possibility of multi-epitope immunotherapy [57]. UB-612 and PepGNP-Covid19 peptide vaccines elicited potent neutralizing antibody and T-cell responses against variants [58, 59].

The PVPredPip (Peptide-Based Vaccine Prediction Pipeline) for SARS-CoV-2 successfully predicted consensus B- and T-cell epitopes with 99.82% population coverage across the globe, showcasing how *in silico* design can aid in pandemic preparedness [19]. Together, these achievements indicate that the peptide technologies are no longer purely theoretically approaches but are moving to an approach with growing translational potential, joining mRNA and recombinant platforms.

During this era of computing (2015-present) consensus pipelines encompassing various algorithms to curb false positives have characterized the period (Fig. 3). Binding and molecular dynamics (MD) simulation-based structural validation provide preliminary structural plausibility checks, population coverage analysis provides vaccine coverage data against worldwide HLA frequency, and AI pan-allelic models provide increased accuracy in underrepresented HLA alleles [60].

This shift from early immunochemical methods to modern AI-based immunoinformatics has bridged the gap between theoretical epitope selection and practical vaccine development. Peptide vaccines are no more hypothetical, rather a proven, licensable platform in addition to mRNA and recombinant platforms for the global response to infectious diseases [57, 61–66].

## Immunoinformatic pipelines in peptide vaccine design

While individual immunoinformatic tools offer important predictions of antigenicity, MHC binding, and population coverage, their greatest impact comes from the incorporation of these algorithms into end-to-end computational pipelines that can design entire multi-epitope vaccine constructions. Such workflows illustrate the application of ‘reverse vaccinology,’ in which whole proteomes are scanned computationally to identify potential regions for antigenic candidates, combined into fragments that can be fabricated as synthetic vaccine entities and subjected to *in vitro* and *in vivo* validation [67, 68].

### The modern computational workflow

Development of modern peptide vaccines uses standardized computational pipelines designed to rank pathogen proteomes for epitopes based on potential immunogenicity. A standard *in silico* pipeline, such as those used in the development of vaccines against ESKAPE pathogens and SARS-CoV-2 will typically follow these general steps [19, 20, 29]:

1. **Antigen discovery and filtering** involve screening whole proteomes for proteins crucial to survival or virulence, and assessed in terms of antigenicity, allergenicity, toxicity susceptibility, and similarity with the human proteome to avoid autoimmunity.
2. **Epitope prediction** for B-cell (linear/conformational), MHC class I cytotoxic T lymphocyte (CTL), and class II helper T lymphocyte (HTL) peptide predictions are executed by popular tools like BepiPred [69], NetMHCpan [70], NetMHCIIpan [71], and IEDB predictors [72].
3. **Consensus building across predictors** by combining outputs from several tools like NetMHCpan, MHCflurry [73], IEDB predictors for T-cell, and multiple structure/propensity methods for B-cell to create a ranked consensus list. This consensus step is widely used by many modern pipelines to boost confidence and cut down on false positives that are exclusive to certain tools. The consensus scoring is done by voting, rank aggregation, weighted averaging, or stacked ML models that learn from the outputs of several predictors. Several pipelines [19, 20, 74] make this an official module. Many published workflows from 2015 to 2025 use ensemble or consensus approaches to improve robustness [75–77].
4. **Population coverage analysis** involves comparison of predicted epitopes to worldwide HLA allele frequency databases to make sure they work for a wide range of people. Analysis of HLA allele frequency is performed to assure high estimated coverage in the analysed dataset, which requires cautious regional interpretation, usually with the target of covering more than 95% of the population.
5. **Molecular docking and MD simulations** involve docking selected epitopes or multi-epitope complexes to MHC molecules and TLRs, followed by MD simulations to evaluate stability and binding affinity.
6. ***In silico* cloning and immunological simulation** entail generated final design is digitally cloned into expression vectors, and computer models simulate immune responses to predict efficacy.

This modular design makes it possible to quickly come up with new vaccine candidates in just a few weeks, instead of the years it would take with traditional methods. This makes it possible to quickly respond to emerging and re-emerging infectious diseases.

### Data infrastructure: the foundation for computational vaccinology

Computational epitope prediction and vaccine design depend fundamentally on high-quality, curated, and equitably representative data resources. The landscape of key immunological databases has evolved significantly, and understanding their strengths, limitations, and complementary roles is essential for reproducible vaccine design [78].

IEDB remains the most widely used and influential resource in peptide-vaccine immunoinformatics because it provides curated experimental data on epitopes, HLA binding, T-cell assays, B-cell assays, and related immune outcomes. However, despite its central value, IEDB alone does not provide a fully sufficient foundation for equitable and reproducible next-generation peptide-vaccine design [79]. Like all curated repositories, it reflects the underlying distribution of experimental attention in the literature, with over-representation of well-studied pathogens, common HLA alleles, and populations for which immunological data are more readily available [80]. As a result, models trained predominantly on such datasets may perform less reliably for rare alleles, under-sampled populations, or emerging pathogens with limited experimental characterization.

For this reason, peptide-vaccine design increasingly relies on the combined use of complementary data resources. Sequence and protein annotation databases, such as UniProt and NCBI, enable antigen selection and sequence retrieval [81, 82]; structural repositories, such as PDB and AlphaFold Protein Structure Database, support conformational analysis and structure-informed modelling [83, 84]; population-frequency resources support more representative HLA coverage assessment [89]; and immunopeptidomics, T-cell receptor repertoire, transcriptomic, and proteomic datasets can provide biologically richer context for prioritizing candidate epitopes. The value of these complementary resources lies not simply in data expansion, but in enabling more auditable, context-aware, and biologically grounded decision-making across the computational pipeline.

Emerging omics resources are also reshaping the field. Mass spectrometry (MS) MHC Eluted Ligand (EL) datasets [85] provide direct evidence of naturally presented peptides and have already improved peptide-presentation models by moving beyond binding affinity alone. TCR Repertoire Sequencing; Single-cell TCR/BCR sequencing and patient-derived repertoire data [86] are beginning to inform personalized vaccine design, e.g. Epi-TAP pipeline [74]. However, TCR reference sets show equity issues, predominantly derived from high-income populations, introducing generalizability concerns for vaccines targeting diverse populations [24, 87].

Nevertheless, expanding the number of resources does not automatically resolve underlying quality concerns. Data incompleteness, uneven annotation standards, inconsistent metadata, differing validation criteria, and limited representation of diverse populations remain important constraints. Consequently, progress in peptide-vaccine immunoinformatics depends not only on larger datasets, but also on better-characterized, interoperable, and transparently reported datasets that are suitable for benchmarking, reuse, and equitable model development [78, 79].

A practical way to compare these resources is through the FAIR framework—Findability, Accessibility, Interoperability and Reusability [88] as shown in Table 2. In the present context, findability refers to stable identifiers and searchable metadata; accessibility to open obtainability of data and documentation; interoperability to machine-readable formats and cross-workflow compatibility; and reusability to licensing clarity, provenance, versioning, and sufficient metadata for downstream reuse.

**Table 2:** A systematic evaluation of bioinformatic resources commonly used in immunoinformatic tools and pipelines against FAIR principles [88].

**Resource**	**Role**	**Findable**	**Accessible**	**Interoperable**	**Reusable**
IEDB [79]	Experimental epitopes for model training and benchmarking	Searchable with stable identifiers (F1, F3) and structure query tools (F4)	Open web access (A1, A1.1) with bulk download and analysis resources	Partial standardization (I1) across assays, ontologies, and some controlled vocabularies used but output format heterogeneity (I2)	Curated experimental annotations (R1.3) support benchmarking, provenance tracked (R1.2), licensing clear for public use (R1.1)
UniProt [81]	Curated proteomes and annotation	Globally unique stable accessions (F1, F3) with highly searchable interface and rich cross-references (F4), comprehensive metadata	Open access (A1, A1.1) through web interface, API, and downloadable datasets, metadata persistent (A2)	Full standardization (I1, I2) through controlled vocabularies and ontologies, qualified cross-references to other databases (I3)	Richly described (R1) with expert curation, detailed provenance (R1.2), meets community standards (R1.3), clear licensing (R1.1)
NCBI [82]	Sequences, genomes, metadata	Comprehensive NCBI search infrastructure (F4) with rich indexing and stable GI/accession identifiers (F1, F3)	Open web interface (A1, A1.1) and programmatic access via Entrez and E-utilities, metadata publicly accessible	Standard formats (I1) (GenBank, FASTA) with some domain-specific limitations, uses taxonomic and functional vocabularies (I2)	Widely reusable (R1), although record completeness and curation depth are variable, clear licensing, community-standard formats (R1.3)
PDB [83]	Experimental structural templates	Stable structure identifiers (F1, F3) with rich structure-level metadata, searchable via sequence, and structure queries (F4)	Open access (A1, A1.1) with downloadable coordinate files and programmatic services (SOAP, REST APIs)	Standard structural formats (PDB, mmCIF) (I1) support integration with modelling workflows, ontology-based annotations (I2, I3)	High reuse value (R1) for experimentally resolved structures, detailed provenance (R1.2), meets structural biology standards (R1.3)
AlphaFold DB [84]	Predicted structural models	Searchable by UniProt accession (F1, F3) with stable identifiers, rich metadata on prediction confidence	Open access (A1, A1.1) with downloadable predicted structures and confidence scores	Standard coordinate-style outputs (I1, I2) easily integrated into structural pipelines, compatible with PDB tools	Reusable for hypothesis generation (R1), predictions include confidence metadata (R1.2), requires context-specific validation
HLA frequency resources [89]	Population coverage/equity	Findability varies by resource (F1-F4), population indexing and dataset granularity inconsistent, no unified identifier scheme	Access uneven across sources (A1, A1.1), differing download and query options, metadata accessibility variable (A2)	Metadata and population descriptors not consistently standardized (I1, I2), limited cross-resource linking (I3)	Useful for equity-aware prioritization (R1), reuse constrained by uneven population coverage and lack of standard provenance (R1.2)
MS-eluted ligand repositories [90]	Experimental T-cell epitopes for model training and benchmarking	Specialized repositories (F1) with limited cross-database indexing (F4), inconsistent searchability	Moderate web access (A1), bulk download options inconsistent across repositories (A1.1)	Partial standardization (I1) of mass spectrometry metadata, limited shared vocabularies (I2), minimal cross-linking (I3)	Curated peptide sequences and MS parameters (R1, R1.3), lacks unified format standards, limited provenance documentation (R1.2)
TCR repertoire resources [86]	Curated TCR-peptide-MHC interaction data for model training and benchmarking	Moderately searchable (F4), usually through sequence, clonotype, or specificity queries, identifier schemes vary (F1-F3)	Access varies across repositories (A1, A1.1), mixed download and licensing conditions, metadata accessibility inconsistent (A2)	Diverse sequence formats and TCR annotation schemas, limited standardization (I1, I2), sparse cross-database linking (I3)	Reusable for specificity modelling (R1), limited by sparse, biased, and unevenly representative datasets, variable provenance documentation (R1.2)

Descriptors are aligned to the FAIR Guiding Principles, focusing on persistent identifiers, metadata richness, searchability, retrieval mechanisms, standards-based representation, provenance, licensing, and fitness for reuse. For FAIR principles (F1-F4, A1-A2, I1-I3 and R1-R1.3), please refer to Box 2 of Wilkinson et al. [88].

A more functional approach is to make data-resource management in peptide vaccinology both routine and auditable. At minimum, all studies should report database versions, query dates, retrieval parameters, and filtering rules, alongside the assay type, evidence strength, and exact criteria used to define positives and negatives [79, 87, 88]. Resource development should also move toward versioned exports with provenance metadata, harmonized identifiers linking sequence, structure, ligandomics, and receptor-repertoire layers, and benchmark-ready datasets released with transparent train, validation, and test splits, leakage checks, and frozen benchmark suites [88, 91, 92]. To address persistent equity gaps, future datasets should deliberately expand representation of under-sampled HLA alleles and geographic populations, with explicit allele- and region-level coverage maps and targeted data generation for African, Asian, and Latin American cohorts rather than relying on global averages [78, 80, 93]. In parallel, the field would benefit from community-agreed metadata minimums, including persistent identifiers, retrieval dates, licensing, protocol details, and cohort descriptors, as well as clearer guidance for assay-specific negative-set construction [53, 88, 91, 94]. Collectively, these steps support the longer-term case for a unified, version-controlled vaccine design data registry that integrates core resources such as IEDB, UniProt, HLA frequency datasets, and MS-eluted ligand repositories under consistent provenance and FAIR-oriented governance [79, 81, 89, 95].

### Vaccine design pipelines

Recent research illustrates the efficacy and constraints of end-to-end computational pipelines. For instance, multi-epitope vaccine candidates for SARS-CoV-2 were developed entirely *in silico* by combining epitopes from spike, nucleocapsid, and membrane proteins. These molecules were docked against human MHC complexes and immune-simulation predictions of strong T-Cell and B-cell responses made [96]. PVPredPip, which exploits this end-to-end workflow, supports such an approach by effectively combining several prediction methods, consensus ranking, and expert curation to produce high-confidence candidates [19]. The pipeline predicted conserved B-cell and T-cell epitopes for SARS-CoV-2 with 99.82% global population coverage estimated *in silico*, underscoring the power of integrated computational design. The approach is thorough; however, the workflow still relies on several standalone bioinformatic tools and involves semi-manual curation steps, which could be a source of error.

Pipelines have also been used to make vaccines against ESKAPE (*Enterococcus faecium*, *Staphylococcus aureus*, *Klebsiella pneumoniae*, *Acinetobacter baumannii*, *Pseudomonas aeruginosa*, and *Enterobacter spp*) pathogens, which are a group of drug-resistant bacteria known to cause infections in hospitals [20]. The pipeline implemented subtractive genomics at the pan-proteome level to mitigate autoimmune risk for ESKAPE infections. It integrated strain conservation filters with docking to innate receptors (e.g. TLRs) and immunological simulations. While this demonstrated the adaptability of bacterial targets to different environments, the approach lacked sufficient experimental validation and transparent reporting.

Epi-TAP [74] went beyond the peptide–MHC interacting capacity and incorporated TCR repertoire recognition using patient-derived sequencing, giving a biological informed triage layer. However, TCR reference sets may be biased towards high-income populations introducing equity issues and reduced generalizability [24].

Recently, DeRoo et al. [97] developed a novel pipeline that utilizes AlphaFold2 [98], named PAbFold to identify linear epitopes to novel antibodies with no structural information available, highlighting recent advancements in linear B-cell epitope prediction. Prediction of conformational B-cell epitopes remains a difficult task due to the complexities associated with accurately identifying discontinuous binding sites based on both structure and sequence [34].

The early success of pandemic technology pipelines such as S-protein multi-epitope SARS-CoV-2 workflows [99] and its implementation of the decade’s dominant architecture (epitope prediction, filtering, population coverage, and docking), often lacked formalized consensus rating or ability to be replicated. Recurrent immunoinformatic pipelines for designing peptide-based vaccine candidates against viruses (Zika, Hendra, Ebola, Monkeypox) [77, 100–102] replicated the same or similar approaches with a different pathogen but without innovation or new external cross validation.

The IEDB benchmarking environment [15] is being used as an underlying evaluation layer for tool performance; however, global HLA allelic gaps are then carried forward and serve to impose fairness limitations on almost all downstream pipelines.

In the case of bacterial targets, subtractive genomics-based pipelines have become routine for reducing autoreactivity by removing host-homologous proteins before predicting epitopes [103–105]. Although such strategies increase the antigen safety, some studies demonstrate that a very strict subtractive filter can lead to the depletion of ‘protective,’ yet subdominant epitopes, reducing the downstream vaccine coverage [106].

Similarly, oncology-targeting peptidomes usually combine advanced experimental validation, e.g. LC–MS/MS immunopeptidomics is utilized to validate natural presenting neoantigens [107–109]. Although promising, these pipelines are not generalizable due to patient-specific mutation profiles, individual HLA heterogeneity, and logistical demands of personalized multi-peptide vaccine production [110].

### Synthesized pipeline derived from over a decade published pipelines

**Figure 4:**
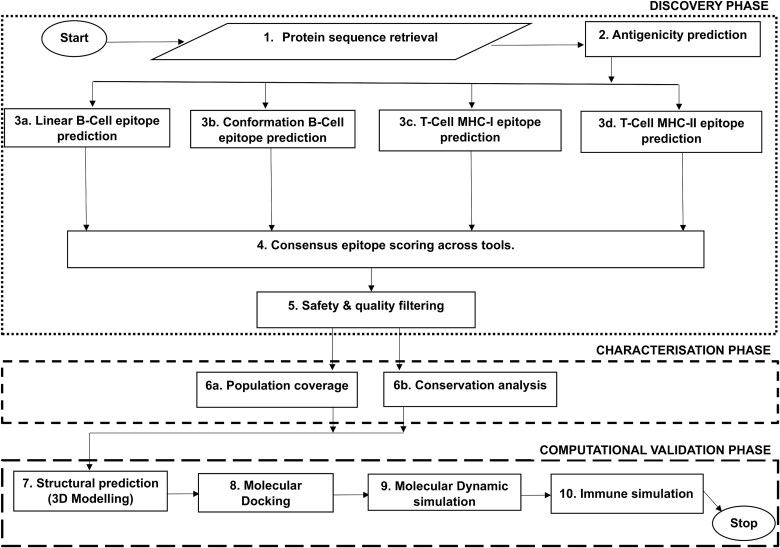
Synthesized computational workflow for peptide-based vaccine discovery derived from over a decade of published immunoinformatic pipelines, illustrating common stages used in modern peptide-vaccine design pipelines. Typical workflows begin with discovery phase which consists of pathogen protein sequence retrieval from protein sequence databases, followed by screening for antigenicity, epitope prediction for T-cell and B-cell responses, consensus prioritization across multiple methods, and screening for safety and quality. Downstream stages commonly include the characterization phase, which involves conservancy and population coverage analysis using HLA frequency data. Finally, the computational validation phase which includes structural modelling, molecular docking, molecular dynamics simulation, and immune simulation of peptide-based vaccine candidates. The workflow summarizes recurrent design stages reported across end-to-end pipelines and is intended as a conceptual synthesis rather than a prescriptive or universally standardized protocol.

The processes shown in the synthesized pipeline (Fig. 4) are examples of broadly conserved design steps that are present throughout the development of peptide-vaccines against pathogens, across platforms and over time. These processes are classified in different phases.

#### Discovery phase

Antigen selection and protein sequence retrieval are essential initial steps, as epitope discovery is inherently limited by the pathogen’s protein landscape; in the absence of a well characterized antigenic source, subsequent computational predictions lack a biological foundation. The common tools in pipelines detailed on Table 3 [75, 101, 111–113] used in this step include NCBI [82] and Uniprot [81].

**Table 3:** Comparative performance of key bioinformatic tools used in peptide-based vaccine pipelines.

**Name of tool**	**Models**	**Training dataset size**	**Test dataset size**	**Reported metrics**	**Strengths**	**Limitations**	**Publication**
**Linear B-cell predictors**							
ABCpred	Recurrent Neural Network (Jordan network)	1400 (700 epitopes and 700 random non-epitopes)	5-fold cross-validation on 1400; Blind sets of 4 proteins and 387 peptides	Accuracy: 65.93%, Sensitivity: 67.14%, Specificity: 64.71%	First systematic use of ANN for BCEs; outperforms individual property scales	Fixed window length (16); high false-positive rate; cannot predict exact boundaries	Saha & Raghava, 2006 [114]
BCEPred	Combined propensity scales (4 properties)	2058 (1029 epitopes and 1029 random non-epitopes)	Evaluated on the training set (2058 peptides)	Accuracy: 58.70%, Sensitivity: 56%, Specificity: 61%	Comprehensive benchmark of existing scales; user-friendly visualization server	Low overall accuracy (<60%); results are qualitative property plots	Saha & Raghava, 2004 [115]
BCPred	SVM with Subsequence String Kernel	1402 (homology-reduced set of 701 pairs)	5-fold cross-validation (1402); Blind set (387 peptides)	AUC: 0.758, Accuracy: 67.90%, Sensitivity: 72.61%, Specificity: 63.2%	Subsequence kernel captures string similarity effectively; provides homology-reduced sets	Performance drops significantly on homology-reduced sets versus redundant data	El-Manzalawy *et al.* 2008 [116]
BepiPred-1.0	HMM combined with Parker’s Hydrophilicity Scale	Positive windows from AntiJen data set (127 proteins)	Pellequer set (14 antigens); HIV set (10 antigens)	AUC: 0.600, Sensitivity: 30.9%, Specificity: 80%	First application of HMM for B-cell epitopes; significantly better than scales alone	Very low sensitivity; major room for improvement in predictive power	Larsen *et al.* 2006 [117]
BepiPred-2.0	Random Forest Regression	155 antibody–antigen crystal structures (3542 positive residues)	5 antigen-antibody crystal structures post-2014; IEDB linear set (30 556 peptides)	AUC: 0.62 (structural), 0.574 (linear IEDB set)	Trained on high-quality structural data from 3D crystal structures	Low overall accuracy for sequence-only tasks; data biased by incomplete annotation	Jespersen *et al.* 2017 [118]
BepiPred-3.0	Deep Neural Network (FFNN) with ESM-2 embeddings	358 antigens (5011 epitope residues, 50% identity reduced)	External test sets of 5 and 15 antigens; IEDB set (3560 sequences)	AUC 0.71 on epitope3D 45-antigen set; AUC 0.738 (TS1), 0.771 (TS2), 0.663 (IEDB)	Uses protein language models (ESM-2) to capture long-range sequence signals	Limited by availability of experimental structures for training	Clifford *et al.* 2022 [69]
COBEpro	SVM based on sequence similarity	Library of positive epitope fragments	10-fold cross-validation on fragment sets; Whole antigens (24 proteins)	Fragment AUC: 0.829, Residue AUC: up to 0.628	Integrated two-step system; can incorporate predicted structural features	AUC drops to ~0.6 when non-epitopes are drawn from same antigens versus random ones	Sweredoski & Baldi, 2009 [119]
LBtope	SVM/K-Nearest Neighbour (IBk)	Lbtope_Fixed (32 652 peptides); Lbtope_Variable (38 197 peptides; Lbtope_Confirm (2837 peptides)	10% independent set	Accuracy: 82.33%, AUC: 0.91	First to use experimentally validated non-B-cell epitopes from IEDB	Fixed-length models still use truncation-extension; redundancy remains a factor	Singh *et al.* 2013 [120]
LBEEP	AdaBoost-Random Forest (ARF) with DDE features	8556 peptides (Original_LBEEP_Dataset)	Independent_Set_1 (949 peptides); Ind_Set_2 (2947 peptides)	Accuracy: 69.97% (Ind_Set_1), AUC: 0.755	Focuses on ‘exact’ epitopes; DDE feature vector outperforms standard dipeptide composition	Information loss observed when under-sampling to create balanced training sets	Saravanan *et al.* 2015 [121]
SVMTriP	SVM with Tri-peptide Similarity and Propensity	9850 peptides (4925 non-redundant pairs)	5-fold cross-validation (9850); Viral/Human tendency test (10^5 peptides)	Sensitivity: 80.1%, Precision: 55.2%, AUC: 0.702	Integrates tri-peptide propensity with Blosum similarity; high sensitivity	Limited to fixed lengths (10–20 AA); Tri-peptides with Q or P bias the model	Yao *et al.* 2012 [122]
BEST	Two-stage architecture using SVM and sliding windows	Filtered40_BCPREDFrag (633 pairs)	SEQ194 (194 antigens); SEQ19 (19 antigens)	Fragment AUC: 0.81, Chain AUC: 0.57 (SEQ194), success rate: 60.3%	First to use residue conservation; fuses multiple inputs (predicted secondary structure, solvent accessibility, similarity to known epitopes)	Chain-based prediction Accuracy is still moderately low (AUC ~0.6)	Gao *et al.* 2012 [123]
EpiDope	DNN (ELMo embeddings and bi-directional LSTM)	~24 600 regions (1798 protein sequence clusters)	10-fold cross-validation; evaluation set (740 proteins, 4767 regions)	ROC AUC: 0.67 (cross-validation), 0.63 (evaluation set)	Uses context-sensitive ELMo embeddings to encode full protein information	Struggles with false-negatives in IEDB short verified regions	Collatz *et al.* 2021 [124]
iBCE-EL	Ensemble of ERT and Gradient Boosting (GB)	Benchmarking set (9925 peptides from IEDB)	Independent set (2518 peptides)	Accuracy: 0.732, AUC: 0.789	First ensemble method for BCEs; consistent performance across benchmark sets	Requires 5-25 AA peptides; dependent on manual curation of assay confidence	Manavalan *et al.* 2018 [125]
DeepLBCEPred	Bi-LSTM and Attention and multi-scale CNN	BCETD555 (1110 non-redundant peptides)	ILED2195 (4390 peptides), IDED1246 (2492 peptides)	Accuracy: 0.77 (cross-validation), 0.67 (Independent set)	Attention mechanism improves interpretability; CNNs capture multi-scale features	Model performance still limited for practical large-scale applications	Qi *et al.* 2023 [126]
EpitopeVec	SVM using deep ProtVec embeddings	Various (e.g. LBTope nr set: 15 677; iBCE-EL set: 9925)	Independent sets (e.g. Blind387, iBCE-EL ind.)	Accuracy: 81.31% (BCPreds set), 75.62% (LBTope set), AUC: up to 0.889	Context-independent protein vectors (ProtVec) encode entire sequence	Generalizability drops substantially when cross-testing across database sources	Bahai *et al.* 2021 [127]
**Conformational B-cell predictors**							
CBTOPE	Machine Learning ensemble	Not explicitly detailed in provided sources	106 glycoprotein antigens	AUC: 0.541 (on glycoproteins)	Convenient when structure is unavailable; uses only primary sequence	Lower performance compared to structure-based methods	Ansari & Raghava, 2010 [128]
DiscoTope-2.0	Weighted sum of log-odds ratios and UHS surface measure	75 antigen–antibody complexes	52 antigen structures (33 homology groups)	AUC: 0.748 (CV); 0.731 (Independent)	Incorporates half-sphere exposure and updated spatial neighbourhood definitions	Does not account for glycosylation; incomplete benchmarks lead to underestimated performance	Kringelum *et al.* 2012 [129]
DiscoTope-3.0	Inverse folding latent representations	Not detailed in provided sources	101 antigens (SEMA 2.0 test set)	AUC: 0.77 (unmasked); 0.716 (masked)	Improved latent representations using inverse folding technology	Not fully detailed in the provided excerpts	Høie *et al.* 2024 [130]
ElliPro	Ellipsoid approximation and residue Protrusion Index (PI)	No training required (geometry-based)	39 epitopes from 39 protein structures	AUC: 0.732 (most significant prediction)	No training needed; handles sequence and structure; strong visualization	Global protrusion estimation can fail for full biological complexes (e.g. multimeric units)	Ponomarenko *et al.* 2008 [131]
SEPPA 2.0	Logistic regression (ASA preference and consolidated AAindex)	314 structures (435 unique epitopes)	42 independent antigen–antibody structures	AUC: 0.745 (baseline); up to 0.823 for mouse-secretory	First to consider subcellular localization and immune host species	Performance remains moderate due to limited known complex structures	Qi *et al.* 2014 [132]
SEPPA 3.0	Logistic regression with micro-environment glycosylation features	767 protein antigens (520 glycosylated)	130 general/106 glycoprotein antigens	AUC: 0.794 (CV); 0.749 on glycoprotein test set	Specifically handles N-linked glycosylation; adjusted false positive rate	Relying on handcrafted features rather than end-to-end differentiable models	Zhou *et al.* 2019 [133]
EPCES	Consensus scoring of multiple surface features	Not detailed in provided sources	49 antigen structures	AUC: 0.695 (in DiscoTope-2.0 evaluation)	Uses consensus scoring on protein surfaces	Performance may be underestimated as it was developed with unbound structures	Liang *et al.* 2009 [134]
EPITOPIA	Naïve Bayes classifier	66 (struct) and 194 (seq) validated epitopes	43 structures (in peer benchmarks)	Struct Success Rate: 89.4%; AUC: 0.60	Bifunctional (struct/seq); powerful visualization; single amino-acid resolution	Naïve Bayes architecture might be over-simplified compared to modern AI	Rubinstein *et al.* 2009 [135]
epitope3D	Adaboost (Graph-based signatures and radius scanning)	180 unbound antigen structures	45 diverse structures (blind test)	MCC: 0.55 (CV); 0.45 (blind test)	Scalable; models epitopes via graph-based signatures; large curated dataset	Experimental structural noises can negatively impact performance	da Silva *et al.* 2022 [136]
GraphBepi	EGNN and BiLSTM (with ESM-2 representations)	577 antigen sequences	56 antigen sequences	AUC: 0.751; AUPR: 0.261	Integrates ESM-2 and AlphaFold2; captures spatial and sequential features simultaneously	Dependent on AlphaFold2 quality; EGNN is largely a ‘black-box’ model	Zeng *et al.* 2023 [137]
ScanNet	Spatio-chemical arrangement of neighbours network	3756 protein chains from SabDab database for B-cell epitope (BCE) prediction	5-fold cross-validation of 3756 protein chains from SabDab database for BCE prediction	AUCPR: 0.178 for BCE; PPV at L/10: 27.5% for BCE	Interpretable filters; end-to-end differentiable; ability to detect conformational epitopes	Performance remained modest; evaluation relied on a limited and incompletely annotated cross-validation dataset	Tubiana *et al.* 2022 [138]
SEMA	Ensembles of fine-tuned ESM-1v and ESM-IF1	~783 non-redundant antigen records	101 antigen sequences	ROC AUC: 0.76	Leverages large pretrained PLM and inverse folding models	Pretrained ESM-1v limit of 1022 residues (longer sequences trimmed)	Shashkova *et al.* 2022 [139]
SEMA 2.0	ESM2-3B and SaProt-650 M ensembles	1544 antigen sequences	101 antigen sequences	ROC AUC: 0.777 (Unmasked ensemble)	Identifies structural similarities; predicts glycosylation; supports multimeric states	Stringent homology filtering slightly reduces metrics for some sub-models	Ivanisenko *et al.* 2024 [140]
**T-cell/MHC predictors**							
TepiTool	IEDB Recommended (Consensus of ANN, SMM, Comblib)	Aggregates data for hundreds of alleles	Varies by underlying method used	Percentile Rank, IC50	User-friendly interface; provides specific guidelines on allele/length selection	A wrapper for existing tools; accuracy depends on underlying third-party methods	Paul *et al.* 2016 [141]
NetMHC-3.0	Artificial Neural Networks (ANN) and PSSMs	55 MHC alleles (ANN); 67 HLA alleles (PSSM)	6452 9-mer peptide affinity data points	Accuracy 75%–80%	Can predict 8, 10, 11-mers using 9-mer trained predictors	Limited to alleles with sufficient experimental binding data	Lundegaard *et al.* 2008 [142]
NetMHCpan-4.0	NNAlign (ANN with insertions/deletions)	>150 MHCs (BA); 55 MHCs (EL, 85 217 entries)	~16 000 ELs; 1251 T-cell epitopes	Not reported	Integrates BA and EL data; captures allele-specific length preferences	Limited EL training data availability at the time of publication	Jurtz *et al.* 2017 [70]
NetMHCpan-4.1	NNAlign MA framework	13 245 212 data points (250 distinct MHC-I molecules)	Independent CD8+ epitope and EL benchmarks	Median FRANK 0.0022; median PPV 0.8291	Uses NNAlign MA to deconvolute and train on multi-allelic (MA) MS data	Inherits MS data biases (e.g. cysteine depletion)	Reynisson *et al.* 2020 [85]
NetMHCIIpan-4.0	NNAlign_MA (with ligand context encoding)	4 086 230 data points (116 distinct MHC-II alleles)	1469 epitopes; 928 neoepitope peptides	Median FRANK 0.0351	Integrates MA data and proteolytic signals; vastly expanded allele coverage	Reduced performance for HLA-DQ alleles due to low ligand counts	Reynisson *et al.* 2020 [85]
NetMHCIIpan-4.1	NNAlign_MA framework	4 086 230 data points covering 116 distinct MHC class II molecules	Independent datasets: 1469 validated T-cell epitopes from the IEDB, 928 neoepitope peptides	Not reported	Broader class II coverage and improved motifs for DR, DP, and DQ	Still limited by uneven class II data availability across loci and populations	Reynisson *et al.* 2020 [71]
MHCflurry 2.0	Neural Network Ensemble and Logistic Regression	493 473 MS entries; 219 596 affinity measurements	Multiallelic benchmark (76 samples)	Not reported	Models allele-independent processing steps separately from binding	Evaluation relying on MS ligands may inflate accuracy due to assay biases	O’Donnell *et al.* 2020 [73]
MixMHCp 2.1	Mixture of Position Weight Matrices (PWMs)	Naturally processed peptides from various cell lines	SYFPEITHI database validation	Not reported	Robustly predicts new HLA properties without existing prediction tools	Motifs must still be assigned to alleles manually or via external motifs	Bassani-Sternberg *et al.* 2016 [143]
MARIA	Multimodal RNN with LSTM	>39 000 unique MS-identified peptide sequences	~3600 ligands from monoallelic K562 lines	Not reported	Integrates gene expression and cleavage signatures; high neoantigen precision	Presentation is necessary but not sufficient for immunogenicity	Chen *et al.* 2019 [144]
MHCnuggets	Long Short-Term Memory (LSTM)	241 553 (Class I) and 96 211 (Class II) data points	BST dataset (23 971 hits); Kim dataset	Not reported	Scalable and fast; single architecture for both MHC classes; handles variable lengths	Does not account for TCR binding or subsequent T-cell activation	Shao *et al.* 2020 [145]
BERTMHC	Transformer neural network with MIL	134 281 data points (Binding); 41 MHC alleles (Presentation)	2413 pairs (Independent BA); 95 638 (Independent EL)	Not reported	Self-supervised pretraining (TAPE) boosts performance with limited data	Based only on peptide/MHC sequences; ignores expression features	Cheng *et al.* 2021 [146]
DeepSeqPanII	RNN (LSTM-CNN) with Attention Mechanism	BD2013 and BD2016 (sourced from IEDB)	BD2016 LOAO (54 folds); weekly benchmark	AUC 0.856	Interpretable via attention; provides insight into binding mechanisms	Supervised methods (e.g. NetMHCIIpan) may offer better core identification	Liu *et al.* 2021 [147]
NetCTLpan	Weighted sum (Binding, TAP, Cleavage)	504 ligand pairs from SYFPEITHI	1695 SYF ligands; 216 HIV epitopes	AUC 0.96	Optimized for high specificity to minimize experimental effort	TAP/cleavage integration may lower performance for sensitivity-focused tasks	Stranzl *et al.* 2010 [148]
**Safety screening**							
VaxiJen	Discriminant analysis by partial least squares (DA-PLS)	100 pos/100 neg (per class: bacterial, viral, tumor)	25 pos/25 neg (external)	Accuracy: 70%–89%	First alignment-free antigenicity predictor; accounts for neighbour effects	Small training datasets relative to modern standards	Doytchinova & Flower, 2007 [149]
ANTIGENpro	Two-stage architecture (40 primary classifiers feeding an SVM)	1324 proteins	External pathogen proteome (*Bartonella henselae*)	Accuracy: 76% (combined); 82% (microarray set)	Homology-free; utilizes unique high-throughput protein microarray data	Earlier versions lacked a web API for large-scale automated pipelines	Magnan *et al.* 2010 [150]
IAapred	Support Vector Machine (SVM)	918 high-antigenicity proteins	218 non-redundant proteins (external)	ROC AUC: 0.761; Sensitivity: 70.2%; Specificity: 70.6%	Open-source and pathogen-agnostic; balanced sensitivity and specificity	Training dataset is heavily skewed toward bacterial proteins	Miles *et al.* 2025 [151]
SAbPred	ABodyBuilder (homology) and i-Patch/EpiPred	SAbDab template databases	AMA-II assessment targets	Paratope: 77% precision at 10% recall	Integrated platform for antibody engineering; provides estimated model accuracy	Requires 3D structural data or high-quality models; not a general safety screening tool	Dunbar *et al.* 2016 [152]
AlgPred	SVM (AAC/DPC) and MEME/MAST and IgE mapping	578 allergens, 700 non-allergens	323 allergens, 101 725 non-allergens	Accuracy: 85% (SVM); 93.5% (combined)	Introduced consensus logic combining multiple prediction strategies	Trained on smaller, older datasets	Saha *et al.* 2006 [153]
AlgPred 2.0	Ensemble (Random Forest and BLAST and MERCI motifs)	10 075 pos/10 075 neg	2015 pos/2015 neg (validation)	AUROC: 0.98	Utilizes the largest current allergen dataset; maps motifs directly to epitopes	Primarily sequence-based; does not explicitly incorporate 3D structure context	Sharma *et al.* 2021 [154]
AllerTOP v2.0	k-Nearest Neighbours (kNN) with E-descriptors	2427 pos/2427 neg	485 pos/485 neg (5-fold CV)	Accuracy: 85.3%	No sequence alignment required; identifies probable route of exposure	Metrics can exhibit high variability compared to newer structural methods	Dimitrov *et al.* 2014 [155]
AllergenFP	Tanimoto similarity of descriptor fingerprints	2427 pos/2427 neg	485 pos/485 neg (10-fold CV)	Accuracy: 88%	Extremely fast similarity search; universal approach applicable to other bio-tasks	Accuracy depends heavily on fingerprint length and resolution step	Dimitrov *et al.* 2014 [156]
AllerCatPro	3D surface similarity and linear window and hexamer	4180 unique allergens	221 pos/221 neg (structural benchmark)	Accuracy: 84%; Sensitivity: 100%	High specificity compared to FAO/WHO rules; models 74% of known allergens in 3D	Limited performance and high runtime for very large (>1000aa) sequences	Maurer-Stroh *et al.* 2019 [157]
AllerCatPro 2.0	Improved 3D and linear and hexamer with autoimmune filter	4979 allergens	218 pos/212 neg (CLICK benchmark)	Accuracy: 84.7%; Sensitivity: 100%; Specificity: 68.9%	Handles nucleotide inputs and very long sequences; predicts ‘low-allergenic’ potential	High computational cost for extensive 3D structure modelling	Nguyen *et al.* 2022 [158]
AllergenAI	Convolutional Neural Network (CNN)	~9588 pos/~9424 neg	~1900 pos/~1900 neg (20% test)	AUROC: 0.97 (test); Accuracy: 0.94	Purely primary sequence-based; automated feature extraction via deep learning	3D structure integration remains in a pilot stage	Liu *et al.* 2025 [159]
AllergyPred	CNN (Proteins)/RF and SVM (Chemicals)	5585 proteins; 1159 chemicals	2993 proteins; 290 chemicals (holdout)	Protein Accuracy: 97%; Chemical Accuracy: 82%	First server for both chemical and biological allergens; distinguishes origin (plant versus animal)	Results are hypotheses requiring further *in vitro* validation	Kemmler *et al.* 2025 [160]
Allermatch	Sliding window (FASTA) and Wordmatch	730 combined sequences	Leave-one-out self-screening	False Negative Rate: 7.5%–23.4%	Directly complies with FAO/WHO Expert Consultation guidelines	High false negative rate for short sequences; limited manually curated dataset	Fiers *et al.* 2004 [161]
ALLERDET	Restricted Boltzmann Machines (RBM) and Decision Trees	4670 pos/4670 neg	763 pos/763 neg (external AlgPred 2.0 test)	Sensitivity: 98.46%; Specificity: 94.37%; Accuracy: 97.26%	Uses RBM for efficient, compact information encoding; maintains high balanced metrics	Performance relies on the initial FASTA feature extraction accuracy	Garcia-Moreno *et al.* 2022 [162]
ToxinPred2	Ensemble (Random Forest and BLAST and MERCI motifs)	8233 pos/8233 neg	1646 pos/1646 neg (validation)	AUROC: 0.99	Optimized for large proteins; highly accurate and robust internal/external validation	Does not classify the specific source or origin of the toxin	Sharma *et al.* 2022 [163]
MultiToxPred 1.0	Ensemble (LightGBM and QDA)	11 041 total sequences (across 27 classes)	2760 independent sequences (test)	Training accuracy 0.840, AUC 0.989; test accuracy 0.846, AUC 0.991	High reported discrimination and broad toxin-class scope	Not peptide-specific and exact train/test counts are not clearly stated in the accessible text	Beltrán *et al.* 2024 [164]

Tools have been grouped based on their task, presenting their underlying models, training and testing data, reported metrics, strengths, limitations, and publication.

T-cell epitope prediction (MHC I and II) is common since the immunogenicity of a peptide is ultimately defined by its binding to MHC, and both CD8^+^ and CD4^+^ responses are necessary for long-lasting immunity. Linear and conformational B-cell epitope predictions are combined because B-cell epitopes are presented either in sequence space as linear epitopes or in three-dimensional structural space as conformational epitopes. Tools commonly used in pipelines outlined in Table 3 [19, 99, 165–167] for linear predictions include Bepipred [69] and ABCPred [114] and for conformational prediction include Discotope [130] and ElliPro [131]. Most pipelines [11, 20, 75, 76, 168] incorporated the consensus scoring which is emerging as a broad but model-dependent requirement to circumvent biases associated with single tool predictions, model assumptions and limits of training data. Overlapping tool predictions make the results more reliable and decrease false positives. The most common tool used for consensus is the Epitope Clustering Analysis - IEDB [15].

Safety and quality filters (antigenicity using Vaxijen [149], allergenicity using AllerTOP [155], and toxicity using ToxinPred [169]) are present in almost every pipeline [19, 75, 76, 102, 165]. This ensures that peptide vaccines do not trigger detrimental or non-protective immune responses.

#### Characterization phase

Analyzing population coverage with Population Coverage - IEDB [15] is also essential given high HLA polymorphism and to ensure that chosen epitopes cover a wide range of ethnic and geographic groupings, directly addressing global equity and versality. Epitope prioritization (conservancy and immunogenicity), as well as structural prediction (including adjuvants, linkers, and 3D Modelling), are necessary to translate short-list predictions into a physiologically feasible vaccine candidate that can trigger coordinated helper, cytotoxic, and humoral immune system responses.

#### Computational validation phase

Structural validation by means of docking (e.g. ClusPro [170] or AutoDock [171]), molecular dynamics (e.g. GROMACS [172]), and binding affinity (e.g. CASTp [173]) is used to ascertain molecular feasibility and to optimize receptor interactions, while immunological simulation (e.g. C-immSim [174]) provides an early estimation of *in vivo* performance. These steps in combination make up a workflow that balances biological relevance, computational robustness, safety, and translational readiness which is why they are increasingly used in different pathogen studies.

Docking, molecular dynamics simulations, and immune simulations can provide useful supportive evidence during candidate prioritization by exploring structural plausibility, interaction stability, and hypothetical immune-response patterns [1]. However, these approaches do not constitute biological validation in themselves. At best, they function as secondary computational filters that may help rank candidates for further study. Experimental confirmation through peptide-presentation assays, binding assays, immunogenicity studies, animal models, and ultimately clinical evaluation remains essential [1].

### Strengths of computational pipelines

The key advantage of these pipelines is their ability to quickly prioritize large number of candidate epitopes. Researchers can prioritize constructs that are most likely to succeed in laboratory validation. This approach saves both time and resources, which is especially significant during outbreaks when rapid response is key. Pipelines also improve global equity by using HLA allele frequency data to make sure that candidate vaccines are made with universal applicability to diverse ethnicities.

*In silico* approaches can model structural interactions (via docking and MD) providing preliminary insights into epitope binding, stability, and immunogenic potential prior to expensive pre-clinical and clinical trials. Modular structure of these also encourages reproducibility, enabling researchers to repeat analyses using new data or tools.

### Limitations and bottlenecks

Despite their potential, existing computational pipelines have several limitations. The variety of tools (Table 3) and their frequent updates compromise reproducibility. Table 3 shows that reported performance cannot be compared naively across tools because evaluation datasets, redundancy controls, epitope definitions, and metrics differ substantially. Cross-validation accuracy on internally curated datasets often overstates real-world utility relative to non-redundant external benchmarks. The table should therefore be read as a structured comparison of model classes, data dependence, and typical strengths/limitations rather than as a simple league table.

Small changes in input processing or the versions of predictors could alter outputs, hindering comparison between studies. Although docking and MD simulations have contributed structural information about such interactions, they may overestimate binding affinities and fail to take into consideration the complexity of an immune response. The absence of experimental validation remains a major limitation as many published *in silico* vaccine designs have yet to undergo lab validation, leaving their real-world efficacy uncertain.

The reliance on human curation of outputs including heuristic filtering and file-format conversions can lead to biased or inaccurate results. This has implications for reproducibility and scalability, particularly during outbreaks when automation is crucial. While recent structure-based and ML methods have shown improved performance, general prediction accuracy is still constrained [33], because of the insufficiency in high-quality antigen–antibody crystal structures; in addition to training datasets bias towards a few extensively studied pathogens, and challenges associated with modelling epitope accessibility and paratope restrictions.

The current models in epitope prediction, many of which are hosted or benchmarked in IEDB [79] may be biased by the availability and distribution of the training data. HLA allele frequencies differ vastly worldwide yet most experimental data are skewed towards alleles prevalent in European ancestry populations. Prediction algorithms tend to work well for these alleles but may have lower accuracy towards more underrepresented HLA types common in Africa, Asia, and Latin America [93]. This has implications for vaccine equity as pipelines that are fine-tuned on partial data may produce epitopes which do not generalize well in low- and middle-income countries (LMICs).

## Artificial intelligence and machine learning in epitope discovery

The integration of deep learning and machine learning into epitope prediction represents the most significant methodological advance in computational vaccinology over the past decade. However, the impact of these tools is constrained by fundamental data limitations and algorithmic assumptions that remain inadequately discussed in the vaccine design literature. This section provides a critical, stratified evaluation of AI/ML contributions while explicitly delineating their current boundaries.

### Pan-allelic MHC binding prediction

Artificial intelligence has substantially improved peptide–MHC epitope prediction, particularly for common alleles with relatively rich training data. Pan-allelic models such as MHCflurry 2.0, trained on convolutional neural networks (CNNs) using both synthetic binding data and mass-spectrometry-eluted ligands, report AUC scores exceeding 0.9 for prevalent HLA alleles like HLA-A*02:01* and *HLA-B*07:02 [70, 73]. By incorporating HLA sequence information, pan-allelic models can extend prediction to alleles with limited direct assay data; however, reliability still depends strongly on representativeness and quality of the underlying training data, and performance often remains uneven for underrepresented alleles. This improved performance represents progress as earlier generation tools (BIMAS, SYFPEITHI) achieved AUC ~0.65 to 0.70, primarily because they relied on limited binding motif databases and could not extrapolate to unseen alleles [176]. Pan-allelic should, however, not be interpreted as bias-free or equally accurate across all alleles. Performance is usually strongest for well-sampled allele families and weakest where ligand and binding datasets are sparse. Model quality should therefore be discussed stratified by allele representation, task definition (binding versus presentation), and benchmark design rather than through global averages alone.

### Structural deep learning for B-cell epitopes

Recent advances in structural deep learning, including AlphaFold-derived structural resources and related modelling approaches such as RoseTTAFold, have expanded opportunities for structure-informed epitope analysis [84, 177], particularly where experimentally resolved protein structures are unavailable. These tools enable conformational epitope exploration, antigen-surface assessment, and structure-aware prioritization. However, their contribution should be interpreted carefully. Predicted protein structures do not directly solve the broader challenge of conformational B-cell epitope prediction, which remains limited by the relative scarcity of high-quality experimentally validated epitope-structure datasets, difficulties in modelling antigen–antibody interaction dynamics, and incomplete benchmarking across diverse antigen classes [33]. Accordingly, structural prediction tools should be viewed as enabling resources within a broader evidence framework, rather than as stand-alone solutions to conformational epitope discovery.

### Interpretable AI and safety-by-design

A critical gap exists between deep learning predictive power and regulatory/scientific acceptance. It is important to understand how and why an epitope is predicted as immunogenic before accepting a model output. This is particularly important for safety assessment, if an epitope prediction tool falsely predicts a cross-reactive antigen that triggers autoimmunity, the consequence is directly patient harm. Software applications like Epitopedia now includes molecular mimicry analysis to help identify autoimmunity hazards [178]. While the utility of deep-learning models for epitope prediction is undeniable, their use in clinical or regulatory decisions has so far been limited by an inherent ‘black-box’ nature.

Interpretability is increasingly important in immunoinformatics because high predictive performance alone is insufficient if the basis of a model’s decisions cannot be examined [179]. Approaches such as attention visualization, feature attribution, and SHAP (SHapley Additive exPlanations) visualization analyses can help identify which sequence, structural, or physicochemical features are most strongly influencing predictions [180–183]. In peptide-vaccine design, such methods may improve confidence in candidate prioritization, support error analysis, and make it easier to identify when a model is relying on spurious patterns or dataset-specific bias [184]. However, interpretability tools should not be treated as proof of biological mechanism; rather, they are best understood as aids for model inspection, hypothesis generation, and transparent decision support.

If a model predicts strong binding for a 9-mer, SHAP can highlight which positions drive the prediction (often anchor residues). Designers can then check whether the highlighted anchors match known HLA motifs, avoid introducing mutations that remove anchors when optimizing constructs, and flag cases where the model relies on implausible residues, prompting conservative interpretation [179]. Attention heatmaps generated by transformer-based epitope predictors can highlight which amino acid positions most strongly influence immunogenicity predictions [182, 183]. While attention mechanisms and SHAP visualizations are valuable for scientific reliance and transparency, they should be positioned as confidence-building measures, not as substitutes for experimental validation. Regulatory pathways must explicitly require transparent uncertainty quantification (e.g. confidence intervals around binding affinity predictions), stratified performance reporting (accuracy for common versus rare HLA alleles), and independent experimental benchmark datasets.

### Rapid multi-epitope design

One of the key bottlenecks in the current pipelines is the manual and step-by-step execution of multiple prediction tools. To solve these challenges, researchers are working on advanced AI frameworks. DeepVacPred, an AI-based *in silico* deep learning approach, replaces multiple predictions and comprehensive evaluations with a deep neural network (DNN) architecture that can rapidly select potential vaccine subunit candidates (reducing the number to around 30) within less than a second, significantly accelerating the design process [185]. However, this consolidation obscures intermediate steps, users cannot inspect epitope rankings before consensus scoring, cannot adjust population coverage thresholds, and cannot understand which binding affinities drove final candidate selection. In regulatory terms, this is problematic, as authorities need transparency about the design rationale, which single-step black-box models inherently obscure [179].

Automation remains attractive because current pipelines are fragmented and often semi-manual. AI-enabled orchestration may reduce turnaround time by harmonizing ranking, filtering and reporting, but faster prioritization is not equivalent to faster licensure. The Next-Generation IEDB Tools platform represents progress toward this goal by enabling users to connect tools together, though fully automated intelligent pipelines remain a key target [95]. Near-term progress is more likely to come from semi-automated workflows that integrate prediction, provenance tracking, uncertainty reporting and benchmark-aware ranking than from fully autonomous systems making unqualified design decisions. These advances suggest that the next frontier will be in automated, transparent pipelines merging ML interpretability with biological plausibility and able to achieve pandemic response thresholds (e.g. CEPI’s 100 Days Mission).

## Current challenges

### Performance gaps in prediction tools

Although epitope prediction techniques have advanced considerably, their accuracy remains limited due to dependency on availability of experimental data of suboptimal quality for training. In T-cell epitope prediction, most tools can distinguish binders from non-binders effectively (often with AUC > 0.9), but struggle to predict absolute binding affinities, particularly for high-potency epitopes [72]. This undermines confidence in candidate rankings. Also, performance is very varied across HLA alleles, with understudied variants demonstrating higher rates of false predictions.

B-cell epitope prediction remains largely unresolved; early benchmarking demonstrated that amino acid property-based methods produce comparable classification outcomes to the random assignment [186]. While recent structure-based approaches have improved, they remain inaccurate, particularly for discontinuous epitopes, which are the most common B-cell binding sites. Beyond HLA-binding to predicting how T-cells respond to antigens, or the more challenging task of TCR-epitope specificity remains a major challenge, and the current techniques perform poorly.

### Performance gaps across HLA diversity

While pan-allelic prediction models have improved, their accuracy still varies substantially across HLA alleles, a problem fundamentally rooted in training data bias. A major limitation is that most of the experimental training data comes from European and North American ethnicities, resulting in underrepresentation of alleles prevalent in LMICs [187]. This sampling bias leads to systematic inequalities where models trained predominantly on one demographic population may not generalize effectively to others.

More specifically, this representation imbalance constitutes a form of aggregation bias, where a model optimized for the overall population fails to account for individual differences across ethnic and gender groups [188]. The result is that despite having representation in training data, underrepresented populations experience substantially worse predictive performance. To address this bias, mitigation strategies should include deliberate efforts to expand training datasets with diverse HLA allele frequencies and to employ fairness-aware classification methods that maximize accuracy across demographic groups rather than optimizing for aggregate performance alone [188].

Benchmarking studies demonstrate that underrepresented HLA alleles have markedly elevated false-positive and false-negative prediction rates, which directly compromise global equity [189]. Computational pipelines may optimize vaccine designs for more privileged groups while neglecting vulnerable ones, an issue frequently articulated in the immunoinformatics and pharmacogenomics fields [186, 189], given the absence of inclusive datasets that represent global HLA variability.

Despite high global coverage estimates, predictions are particularly low for South Africa, which concurs with previous findings that the IEDB population coverages estimates using South African cohorts have been poor [19, 190]. Several studies have proposed credible causes, including insufficient or obsolete HLA frequency information for African populations inside the IEDB Population Coverage Tool [191, 192]. High allelic diversity and rapid HLA turnover within sub-Sahara populations results in decreased overlap with epitopes optimized for globally prevalent alleles [192]. Global training set bias exists due to limited availability of experimental epitope binding data for African prevalent alleles [189].

Recent studies continue to highlight this discrepancy. Research on multi-epitope vaccines for schistosomiasis, hepatitis C, and SARS-CoV-2 frequently indicates significantly reduced IEDB coverage in South Africa and other African areas, despite global coverage exceeding 90% [96, 193]. These findings collectively highlight the structural deficiencies in existing datasets and the necessity of integrating various HLA repertoires, notably African allelic diversity, into next generation immunoinformatic pipelines.

### The conformational epitope problem

Most protective antibody responses target conformational B-cell epitopes, which are discontinuous residues brought together by protein folding. Recently developed prediction tools exhibit low prediction accuracy due to poor structural training data, computational intricacy in modelling antibody–epitope interfaces and lack of established benchmarks [33]. Until these challenges are addressed, rational antibody-based vaccine design will remain restricted, thereby limiting the promise of peptide vaccines for neutralization-dependent protection.

### Data quality, bias, and reproducibility

ML models are limited by the quality of their training data, which is typically sourced from centralized repositories such as IEDB. This data is also biased; for example, HLA alleles are unevenly represented, with most coverage from those common in European populations. Studies comparing antibody repertoire analysis techniques report discrepancies in germline gene databases and annotation methods can significantly alter results [92].

Curating data for submission to public databases can be a long and tedious procedure involving complicated templates and logistical challenges. For end-users, making complete predictions means using multiple different tools and putting the data together manually, which is inefficient and can lead to errors [178]. It is difficult to reproduce results over time as AI models, and their databases are rapidly evolving. This is a major problem that developers of programs like PAbFold employing AlphaFold2 [97] have to navigate.

Disparities in data pre-processing, tool versions and parameter settings can lead to discrepancies in predictions for the same pathogen sequence. Most pipelines published are not version controlled or reproducible; confidence in results is restricted and progress toward regulatory acceptance hampered. For reproducibility, pipelines should include version pinning, standard operation procedures, and provenance tracking.

### Translation and validation bottlenecks

Most *in silico* vaccine designs are yet to progress beyond the publication stage, with a minority advancing to laboratory or clinical validation. For example, multi-epitope SARS-CoV-2 peptide vaccines UB-612 and EPV-CoV19 which were advanced into phase I/II trials [58, 194], and computationally prioritized universal influenza candidates have proceeded to preclinical evaluation [195]. These are rare instances of candidate progression, compared to the number of published candidate designs. This gap exists because *in vivo* and *in vitro* testing, preclinical evaluation, and clinical trials take a lot of resources and time to move from computational design to production.

Although *in silico* analysis suggests a possible strong protective immunity, experimental confirmation through *in vitro* and/or *in vivo* investigation is necessary to robustly validate the protective immunological efficacy of proposed candidates. The difficulty is in setting up high-throughput confirmation and validation systems that can keep pace with the speed of computational predictions. Further development of confirmation and validation systems will ensure that only the most promising candidates are efficiently advanced to clinical development.

### Regulatory and safety considerations

It is worth noting that existing regulatory paradigms were developed for antigen and platform-based vaccines and not AI-predicted components. To ensure computational workflows follow established safety and effectiveness requirements, clear reporting interpretable AI models, and regulatory sandboxes where new ideas can be tested are required [196].

Regulatory frameworks necessitate explicit rationales for candidate selection, especially when computer outputs directly influence pre-clinical prioritization. AI-driven technologies that produce interpretable results, like evidence of specific peptide–MHC interactions or structural motifs, are more likely to be accepted by both the scientific community and regulators. Ethical monitoring must encompass not only efficacy and safety but also global representation, data governance, and equal access.

### Formulation and manufacturing challenges

Nowadays, there is no guarantee even when computational epitopes are correctly discovered that the vaccine candidates created are safe and work well *in vivo*, as adjuvants and delivery mechanisms also need careful consideration. Innovation is limited by lack of global acceptance of new adjuvants, and scaling the production is more challenging due to different antigens having varied effects on formulations. Peptide vaccines have been slow to reach the market, mostly because people are worried about the safety of the adjuvants that go with them, not because the antigen itself does not work [197].

The difficulty of quickly turning new vaccine candidate findings into standard immunization methods that can be used by many people still exists. Complexity in scale-up and quality assurance of the multi-component formulations as well as high reactogenicity of potent adjuvants leading to local or systemic inflammation may compromise tolerability. Even when computation design is successful, manufacturing complexity is still a major hurdle for the large-scale vaccine deployment.

## The role of formulation in vaccine design

While identifying the right epitopes by computer modelling is the first step, formulation determines the success of the vaccine. To provide protective immunity, synthetic peptides need strong adjuvants and delivery mechanisms. TLR agonists (CpG, poly I:C, MPLA) as adjuvants stimulate innate immunity and promote Th1 responses necessary for CTL priming [49]. Nanoparticle carriers, including liposomes, virus-like particles, and self-assembling peptide nanofibers, safeguard peptides against degradation while specifically targeting antigen-presenting cells [198]. It is important to distinguish peptide antigens from the delivery or formulation systems used to present them. Nanoparticles, carrier proteins, adjuvants, and related technologies may enhance peptide-vaccine performance, but they are formulation or delivery strategies rather than equivalent antigen-platform categories.

Peptide vaccines remain stable at high temperatures for months on freeze-dried formulations and do not need to be kept cold making them crucial for efficient and equitable global distribution. Recently, Q11 peptide scaffolds have shown preserved structural integrity and immunogenicity with storage at 45°C, surpassing traditional adjuvanted vaccines [199]. This inherent durability makes peptide vaccines suitable in low-resource regions where cold-chain infrastructure is not readily available.

## Lessons from cancer and infectious diseases

### Cancer vaccine insights

Despite being initially designed for infectious diseases, significant clinical development of peptide-based vaccine concepts has taken place in oncology. Tumour present a special environment in which tumour-associated or tumour-specific antigens can be targeted by the immune system via SLPs. Although clinical responses have been variable, the collective data offer important design, formulation, and immunological concepts of equal value to infectious disease vaccinology.

Early studies used mutant RAS-derived peptides aimed at inducing CTL responses against oncogenic drivers. However, despite detectable T-cell responses in some patients, responses were low, short lived, and clinically ineffective [44]. A key limitation of these minimal peptides was their inability to attract CD4^+^ T-cells.

Follow-up SLP vaccines against MUC1 and HER2 were much more immunogenic than minimal peptides in the presence of Montanide + TLR agonists [200]. These studies demonstrated that, for peptide vaccines to give rise to potent and lasting responses, helper epitopes are required, a concept which is now believed to be foundational in the designing of peptide vaccines.

The emergence of next generation sequencing further facilitated production of personalized neoantigen vaccines, designed against an individual’s tumour mutations. Trials with synthetic long peptides that carry the neoepitopes of individual patients have also provided additional preliminary evidence of T-cell responses and early signs of clinical efficacy [107]. These experiments demonstrate the potential to combine sequencing-based epitope discovery pipelines with peptide synthesis and vaccine preparation. A similar approach could be used in infectious disease scenarios where sequence analysis of pathogen genomes enables reflection on rapid computational vaccine design.

The clinical results continuously prove that peptide monotherapy is insufficient to complete cancer remission. Long-term tumour regression with current therapies typically requires combination of immune checkpoint inhibitors (anti-PD-1, anti-CTLA-4) or other immunomodulatory agents.

Evidence from therapeutic cancer peptide-vaccine studies is informative for understanding design logic, delivery strategies, immunogenicity challenges, adjuvant use, and combination approaches, but it should not be interpreted as direct proof that infectious-disease peptide vaccines have reached equivalent translational maturity. Oncology and infectious disease vaccine development differ in target biology, immune context, efficacy endpoints, and clinical objectives. Cancer studies, therefore, provide useful translational lessons, but their significance lies primarily in informing platform development rather than in demonstrating broad readiness for infectious-disease deployment.

### Infectious disease successes

In infectious disease settings, peptide-vaccine development has shown encouraging progress, particularly in early-stage preclinical and clinical studies where immunogenicity, safety, and candidate prioritization have been demonstrated for selected pathogens. These findings support the broader feasibility of peptide-based approaches, but they do not yet indicate that peptide vaccines have achieved routine, large-scale clinical deployment across infectious disease indications. Current evidence is better interpreted as proof of growing translational potential rather than as confirmation of full platform maturity [57]. Early peptide vaccine candidates show promise. UB-612, a multi-epitope vaccine for COVID-19, exhibited robust neutralizing antibody and T-cell responses across viral variations, supporting the computational epitope selection methodologies [65, 66].

Synthetic long peptide vaccines have effectively elicited robust T-cell responses and neutralizing cross-reactive antibodies for malaria [61]. The J8-diphtheria toxoid peptide vaccination for Group A Streptococcus has shown efficacy in preliminary clinical studies [63]. HIV research has utilized lipopeptide vaccines that have shown safety and efficacy in clinical studies [64].

Several peptide vaccines are registered at different stages of clinical trials (Phases I–IV) for a variety of diseases such as HIV, cancer, hepatitis B/C, influenza, malaria, and COVID-19. This cross section of candidates reflects the increasing confidence on peptide-based methods and their move from on-paper constructs to viable, scalable clinically validated vaccine platforms.

## Global preparedness

### The 100 days Mission

Preparedness requires not only innovative science but also equitable global benefit-sharing. The CEPI’s 100 Days Mission and the WHO’s R&D Blueprint highlight the importance of open data, platform interoperability, and equitable vaccine access during outbreaks [12, 13]. These initiatives set the ambitious objective of developing, testing, and mass producing an effective vaccine within 100 days which is currently unachievable with existing methods.

To achieve such a goal demands novel innovative platform technologies that support sequence-to-candidate pipelines. AI-enhanced immunoinformatic pipelines can substantially accelerate the early-stage identification and prioritization of candidate epitopes after pathogen genome release, potentially compressing parts of the discovery timeline from months to days. However, their performance remains constrained by data quality, dataset imbalance, limited benchmark standardization, and persistent underrepresentation of many HLA alleles and population groups. Moving towards this vision will require tackling present bottlenecks in reproducibility, HLA ‘representation’ and the ability to validate experiments. Rapid computational design should not be conflated with biological validation or clinical readiness, both of which still require iterative experimental and regulatory evaluation.

### FAIR data principles and minimum reporting standards for *in silico* peptide-vaccine pipelines

Achieving rapid, reproducible, and globally relevant peptide-vaccine design requires a data ecosystem that is Findable, Accessible, Interoperable, and Reusable (FAIR) [88]. IEDB represents an important example of this approach, but broader adoption of FAIR principles across immunoinformatic resources remains incomplete [79]. Proprietary algorithms, inaccessible training data, inconsistent metadata, locked workflows, and limited reporting of negative datasets or preprocessing steps continue to restrict reproducibility and independent validation. In practice, this means that apparently similar pipelines may generate different outputs without a clear audit trail, making comparison, replication, and regulatory confidence more difficult. Strengthening FAIR implementation across datasets, tools, and reporting practices (Table 4) is therefore not a technical preference alone, but a strategic requirement for preparedness-oriented peptide-vaccine development [201].

**Table 4:** Proposed minimum reporting standards for *in silico* peptide-vaccine pipelines.

**Reporting domain**	**Minimum information to report**
Data provenance	Exact database versions, access dates, accession identifiers, assay inclusion criteria, negative-set definition, and deduplication/class-balancing rules.
Sequence/structure inputs	Reference proteome build, strain/isolate IDs, sequence filtering rules, structure source (PDB versus AlphaFold/RoseTTAFold), and confidence thresholds.
HLA layer	Allele list, nomenclature standard, HLA frequency source, region/population used, handling of rare alleles, and whether coverage is reported globally and regionally.
Prediction tools	Tool name, version, mode, parameters, thresholds, and whether defaults or custom settings were used.
Training/benchmark context	Dataset origin, train/validation/test split logic, external validation status, and metrics used (AUC, PR-AUC, PPV, recall, calibration where available).
Consensus strategy	Voting, averaging, stacking or rule-based aggregation logic and tie-breaking rules.
Filtering steps	Antigenicity, allergenicity, toxicity, autoimmunity/homology, conservancy, and immunogenicity thresholds.
Structural assessment	Docking engine/version, receptor template, peptide preparation, scoring functions, MD engine/force field, simulation time, restraints, and convergence criteria.
Intermediate outputs	Share ranked peptide lists before and after each filter, not only the final construct.
Code/environment	Version control repository, executable workflow where possible, environment specification, random seeds, and archived release DOI.

To support reproducibility, comparison, and eventual translational confidence, *in silico* peptide-vaccine studies should report a minimum set of methodological details (Table 4). These include the exact source of all sequence, structure, HLA-frequency, and immunological datasets; accession numbers and retrieval dates; inclusion and exclusion criteria; preprocessing steps; negative-set construction; train, validation, and test split strategies; tool names, versions, and parameter settings; decision thresholds; external validation datasets where used; and the intermediate outputs generated at each stage of the pipeline [202]. Studies should also report whether data leakage was assessed, how HLA alleles were selected, whether population-frequency data were incorporated, and whether code, scripts, or workflow environments are available for reuse [175, 203]. Such reporting does not eliminate biological uncertainty, but it substantially improves transparency, interpretability, and the ability of other researchers to reproduce or benchmark findings.

### Addressing HLA diversity and population equity

A significant hurdle in the development of peptide-based vaccines is the inadequate representation of HLA alleles from LMICs in epitope prediction datasets. Algorithms typically show higher prediction accuracy for alleles which are common in European populations. This means that their predictions may not hold true for all population groups in a global context [33, 189]. This limitation could lead to patterns of exclusion where new vaccinations are made to work best for some groups while others stay at risk, undermining equitable access to safe effective vaccines, regardless of ethnicity or socioeconomic status.

Efforts to increase the size of global HLA frequency databases and incorporate different data sets in training algorithms, along with the prioritization of pan-allelic models are essential to rectify this [204]. AI methods can generalize across alleles, but without representative data, predictive equity may not be guaranteed. Therefore, global data-sharing programs are not only scientifically required, but also ethically necessary for truly equitable pandemic preparedness.

## Future directions

### AI-powered pipelines

Next generation immunoinformatic pipelines should combine advances in AI with mechanisms for fairness, bias detection, and reproducibility during all stages of model development. Beyond leveraging successes of present methods improved by AI and standardized principles, fair data integration is essential, including adoption of FAIR-compliant data layers (Findable, Accessible, Interoperable, Reusable) [88, 187]. This infrastructure enables tracking of data lineage, version consistency, and reproducibility across labs, all of which are critical for detecting and preventing the propagation of biases across institutions.

Additionally, achieving fairness in clinical AI systems requires pre-processing interventions to remove biases from data, in-processing fairness constraints during model training, and post-processing fairness evaluations before deployment [188]. Specifically, bias can originate during data collection through admission bias, volunteer bias, or observer bias, leading to non-representative samples that lack diversity [205]. By implementing FAIR data standards alongside formal bias assessment and fairness metrics before clinical deployment, pipelines can substantially reduce the risk of perpetuating or amplifying algorithmic inequities.

The creation of current epitope-based vaccines depends on public immunological databases like the Immune Epitope Database (IEDB) [79] and UniProt [81]. It also uses different online servers for analysis [113, 206]. The need for next-generation pipelines to incorporate standardized data layers in accordance with FAIR principles is a direct manifestation of the current reliance on consistent, quality data input and cross-validation across various tools [17, 72].

Modern vaccinology relies on machine learning algorithms, such as Support Vector Machines (SVM) and Artificial Neural Networks (ANN) [103], to predict key parameters including peptide binding affinity and immune response. In future, deep learning can be used to improve these prediction models for all key HLA alleles, improving the capacity of models to generalize across a broader range of alleles, while acknowledging that performance remains dependent on the quantity, quality, and representativeness of the underlying training data. It is also important to make equity-aware dashboards that show HLA coverage since the effectiveness of epitope-based vaccines depends a lot on how well the chosen epitopes bind to the highly polymorphic MHC/HLA molecules found in different human populations. Current studies already use mandatory population coverage analysis to make sure that the vaccination is effective across a large portion of the world’s population (Fig. 4).

To make predictions more accurate for groups that are not well represented, there is a need for pan-allelic deep learning architectures that are trained on MHC ligand datasets from all over the world. New transformer-based and graph neural network models are showing promise for modelling epitope–MHC interactions with unparalleled accuracy [207]. However, future versions need to include equity-aware training processes that fix HLA frequency imbalances and gaps in geographic data. Also, built-in explainable AI features like attention heatmaps, residue-level attribution, and model cards will address the black-box nature of complex algorithms by explaining epitope selection rationales [208]. This will make people more likely to trust the computational prediction. Automated reporting pipelines that can make summaries that are ready for regulatory review (such as performance metrics, allele coverage profiles, and uncertainty estimations) could shorten review cycles significantly [209]. Integrating these solutions together would shorten the time it takes to go from design to candidate from weeks to hours, making openness, fairness, and producibility key characteristics.

### Integration with emerging technologies

In the future, immunoinformatic pipelines will work more closely with new experimental and structural technologies. This will make it easier for computation and wet-lab validation to work together. Enhancements in structural prediction tools beyond AlphaFold [97, 98], aimed at integrating dynamics, antibody–antigen interactions, and immunogenic conformations, will significantly improve the precision of conformational B-cell epitope prediction, which is often essential for eliciting robust humoral (antibody) immune responses. Single-cell multi-omics (transcriptomics, TCR/BCR sequencing, chromatin accessibility) can provide personalized mapping of immune repertoires, hence enabling the development of individualized peptide vaccines that target predominant T-cell clonotypes [210].

Synthetic biology principles are used to develop Multi-Epitope Vaccines (MEVs) from the ground up [211]. These designs use specialized linkers (like EAAAK, GPGPG, AAY, and KK) to maintain the B-cell and T-cell epitopes structural independence, making them more stable, and stop the formation of non-native ‘junctional’ epitopes [103]. Also, adjuvants like defensin, flagellin, or RS09/PADRE sequences are added to the peptide platform with linkers like EAAAK to make it more immunogenic by activating both innate and adaptive immunity through TLR binding [212, 213]. Future synthetic biology innovations seek to improve the intrinsic stability of these peptide substrates, occasionally investigated through methods such as disulfide bond engineering [214]. Synthetic biology will provide new types of programmable peptide scaffolds that are more stable at high temperatures, can self-assemble, and have built-in adjuvanticity to unify both the design and formulation [197].

In immunoinformatics, current computational prediction is speedy, useful, and inexpensive, but its applicability is limited to the *in-silico* environments. High-throughput microfluidic and droplet-based systems that can screen hundreds of peptides-MHCs or peptides-antibodies interactions every minute will allow experimental validation to keep up with computational prediction [215]. For example, rapidly evolving interaction-prediction approaches, from structure-informed binding frameworks to graph-enhanced large language models for antibody–antigen affinity prediction, highlight a future direction in which increasingly sophisticated AI models can refine immune interaction mapping and candidate prioritization [216, 217]. Finally, systems vaccinology methods that combine omics data, computational immunology, and mathematical modelling will make it possible to find correlates of protection early on, speeding up the process from candidate selection to Phase I trials [218]. Collectively, these technologies will change the way peptide vaccines are developed from a linear pipeline to a real-time, iterative, and data-driven ecosystem.

### Universal vaccines against evolving pathogens

With continuous evolving pathogens due to natural immunity and vaccination, it is important for future pipelines to focus on making universal vaccines that can defend against a wide range of variations, groups, and species [219]. Artificial intelligence-based evolutionary models are becoming available to dissect sequence diversity among thousands of viral sequences, finding conserved epitopes that are functionally constrained because they have a fundamental role in the viral life cycle such as replication, entry, or immune evasion [220]. Targeting such regions, for example receptor-binding motifs, fusion peptides, polymerase active sites, or structural hinge regions minimizes the chance of immune escape and maximizes cross-strain coverage [221], providing enduring, universal protection.

Deep mutational scanning data can be integrated into predictive models to rank epitopes not only by immunogenicity but also by evolutionary escape potential, enabling prioritization of targets with low mutation tolerance [222]. For high mutation viruses (e.g. influenza, HIV, SARS-CoV-2), multilayer epitope assembles combining conserved T-cell epitopes, structurally constrained B-cell epitopes, and cross-reactive helper regions may generate vaccines which remain effective over extended time [223]. Universal peptide vaccines offer a strategic direction for long-term pandemic preparedness minimizing dependency on seasonal updates and improving adaptability to future outbreaks.

## Conclusion

Peptide-vaccine immunoinformatics has evolved from relatively simple sequence-based screening into a multi-layered computational workflow that increasingly incorporates structural modelling, machine learning, population-aware analysis, and translational planning. This progress has significantly improved the speed and sophistication of early-stage candidate discovery. However, major barriers remain, including uneven data quality, limited benchmarking standards, poor reporting consistency, underrepresentation of HLA alleles and some population groups.

The most promising near-term pathway to clinical adoption is therefore unlikely to come from ever more complex black-box prediction alone, but from benchmark-aware, semi-automated, and transparently reported workflows that integrate diverse data resources, structure-informed modelling, equitable HLA coverage, and early experimental feedback. In this context, AI should be understood not as a replacement for biological and clinical validation, but as a powerful accelerant for hypothesis generation, candidate prioritization, and preparedness-oriented vaccine discovery. If supported by stronger reporting standards, FAIR data practices, and translationally grounded validation frameworks, peptide-vaccine immunoinformatics can make a meaningful contribution to rapid-response goals such as CEPI’s 100 Days Mission. Overall, the field has made substantial methodological progress, but future impact will depend less on isolated gains in prediction accuracy and more on transparent, reproducible, equitable, and experimentally grounded workflows that can support real-world vaccine translation.

## Data Availability

The data underlying this article are available in the article.
